# ‘Total Pain’ in Children with Severe Neurological Impairment

**DOI:** 10.3390/children5010013

**Published:** 2018-01-18

**Authors:** Timothy A. Warlow, Richard D.W. Hain

**Affiliations:** All Wales Paediatric Palliative Care Managed Clinical Network, University Hospital of Wales, Heath Park, Cardiff CF14 4XW, UK; richard.hain@wales.nhs.uk

**Keywords:** ‘Total Pain’, paediatric palliative care, chronic pain, persistent pain, cerebral palsy, cognitive impairment, neurological impairment

## Abstract

Many children with palliative care needs experience difficulty in managing pain. Perhaps none more so than those with severe neurological impairment. For many years; behaviours in these children were misunderstood. As a result; pain was poorly recognised and inadequately managed. Significant advances have been made in the assessment and management of pain in this challenging group of patients. We summarise these advances; drawing on our own experience working with infants; children and young adults with palliative care needs within a UK tertiary paediatric palliative care service. We expand on the recent understanding of ‘Total Pain’; applying a holistic approach to pain assessment and management in children with severe neurological impairment.

## 1. What Is ‘Total Pain’?

The internationally accepted definition of pain is:

An unpleasant sensory and emotional experience associated with actual or potential tissue damage, or described in terms of such damage (International Association of the Study of Pain (IASP) 1973).

[1]

This definition makes two specific points. Firstly, the pain experience is an inherently subjective one. It can only be described accurately by the person experiencing it, and sometimes not even by them. Secondly, pain is not just a physical experience. The relationship between physical tissue damage and patient experience of pain is highly complex. Long gone are the days when pain was described as in a purely linear relationship with nociceptive input. With our understanding of the complex integration of these inputs with cortical networks, and supraspinal modulation of pain through descending pathways, even suggestions of pain as either mental or physical are a thing of the past [2]. Cicely Saunders in the 1960s saw a broader view than the IASP definition, acknowledging the impact of spiritual and psychosocial aspects on the pain experience, coining the term ‘Total Pain’ [3]. Pain is experienced as an overall feeling state with multiple layers of meaning. This is particularly true for children with palliative care needs, for whom the onset of pain may represent the relentless progression of a life limiting or life-threatening disease. Table 1 lists some of the key factors identified as impacting the experience of pain in children.

## 2. Total Pain in Children with SNI

For children with severe neurological impairment (SNI), our definition of pain becomes fraught with difficulty. How can we assess and manage the individual pain of children who cannot verbally communicate their experience? When cognitive appraisal is the most significant factor in the affective and spiritual dimension of pain, how can we understand their unique experience in order to holistically assess and manage their pain? [3].

### 2.1. Physical Aspects of the Pain Experience

90% of children with SNI experience recurrent pain for more than a year during childhood [5]. For 75% this is on at least a weekly basis and for 50% the pain episodes last longer than 9 h [6]. These children experience more episodes of nociceptive pain and a greater number of pain sources than those with mild to moderate impairment [6]. Table 2 highlights some of the sources of pain identified in children with SNI.

Children with SNI experience pain more intensely than children with normal neurological function. They are also vulnerable to intensely distressing episodes of pain without identifiable cause, and abdominal pain despite optimal treatment of constipation and gastro-oesophageal reflux disease (GORD). In addition to the breadth of nociceptive sources of pain, there are three physical processes contributing to this increased pain experience, all with their origins in the central nervous system (CNS).

#### 2.1.1. Central Neuropathic Pain

Riquelme et al. studied proprioception, touch and pain pressure thresholds in 15 children with cerebral palsy and found significantly increased sensitivity to painful stimuli. Children with CNS damage display altered excitability in the somatosensory cortex [5,8]. Nociceptive processing at a molecular, cellular and circuit level are altered, leading to system wide changes in neuroexcitability that ultimately lead to an amplified pain experience [5].

#### 2.1.2. Autonomic Dysfunction

Damage to the autonomic nervous system is common in children with SNI. It can result in abdominal pain, retching and constipation, due in part to gastrointestinal dysmotility [5]. Key features include flushing or pallor, heart rate changes, increased saliva production alongside abdominal symptoms.

#### 2.1.3. Sensitisation to Pain

Children with SNI may have experienced many painful procedures early on in life, from blood tests on the neonatal unit, through to invasive investigations and surgery. GORD, constipation, and insertion of gastrostomy tubes also provide repeated mechanical and chemical stimulation to what is already in many children a dysmotile gastrointestinal tract. These repeated nociceptive inputs lead spinal afferent neurones to become sensitised peripherally [9,10].

Nociceptive signals from sensitised spinal afferents are repeatedly received at the dorsal horn of the spinal cord. This can lead to a progressive build-up in amplitude of action potentials and cumulative depolarisation known as the ‘wind-up phenomenon’ [11,12]. As nociceptive fibres continue to be activated, children experience a progressively intense sensation of pain, out of proportion with the original stimulus. In addition, low-level signals from peripheral nociceptors can lead to increased synaptic efficacy of spinal cord neurones. As a result, exaggerated nociceptive signals are produced long after the pain stimulus has gone. Non-nociceptive input from other neurones, (such as those produced by normal gut movements or light touch), become amplified, and trigger action potentials in spinal pain pathways. The resulting process of central sensitisation is one of disproportionately widespread pain which persists for longer and at greater intensity than is expected from the original stimulus [5,11,13]. Visceral hypersensitivity describes these peripheral and central sensitisation processes when the stimulus originates in from the body viscera, especially the gastrointestinal tract.

A further degree of modulation occurs at the point of cognitive and emotional processing, leading to interpretation of non-noxious sensations as noxious. This leads to hypervigilance [14]. This can amplify the pain experience further. It therefore becomes clear that even at a neuronal network level, psychosocial and spiritual factors are fundamentally entwined with physical aspects of pain.

Contribution from these neuropathic elements is suspected when children have higher baseline pain ratings and significant intensity and duration of pain attributed to experiences that are not normally painful. In children with abdominal pain, features include a history of pain with tube feeds, bowel gas and before bowel movements. Pain relieved by slowing or cessation of feeds, or substitution of feed for electrolyte solution is also suggestive [5].

In addition to the above, seizures, dystonia and contractures due to spasticity may cause pain, be triggered by pain from other sources, or become involved in the expression of pain behaviours. In reality, most children with SNI have more than one cause of pain, and a mixture of nociceptive and neuropathic pain elements [5].

### 2.2. Psychosocial and Spiritual Aspects of the Pain Experience

It is clear that the perception of pain is a function of a child’s cognitive and emotional development [1]. While affective and spiritual dimensions of suffering must depend to a certain extent on cognitive ability, it would be wrong to assume that only patients with fully developed cognition can experience existential distress. Children with SNI are in fact more susceptible to psychosocial problems. There is an increased prevalence of emotional and behavioural difficulties that significantly impact the quality of life for them and their families. In a study by Dolapo et al. of 22 children with dystonic cerebral palsy, they were found to have more difficulty understanding their own mental states when compared to the group of 20 control subjects. In addition, they had a reduced ability to manage and monitor their emotion [15]. Patients, especially those who are very young or who have a more significant degree of cognitive impairment, may perceive pain to be a form of punishment, and find it difficult to rationalise its cause, recognise that it will come to an end or anticipate the impact of analgesia. In children with SNI, chronic or recurrent pain can lead to outburst of aggression, withdrawal from the world socially, reduced adaptive abilities in communication, and have a significant impact on function in daily life [1,6,16]. Emotionally they may have little in the way of coping strategies [1]. Fear, sadness and anger are the dominant emotions [1]. These factors all amplify the pain experience.

Spirituality for the child can be best defined as ‘how they make sense of the world and their place in it’ [16]. The spiritual experience of pain in children with SNI is also therefore grounded in their cognitive and emotional development. James Fowler suggests that for children of a developmental age akin to those with SNI, an appreciation of meaning is made from the bonds of attachment and mutual relationship experienced by the child [16]. The ability of the parent or carer to appraise the experience of the child and respond appropriately makes it possible for the child to trust and therefore hope, a definitively spiritual concept [4]. Children with SNI may be limited in their ability to give signals and respond reciprocally to the signals of others [2]. Atypical behaviours in response to pain may be misinterpreted or unrecognised by caregivers. This breakdown in mutual experience between the child and carer can lead to existential distress, further exacerbating the pain experience [2]. Children with SNI are completely reliant on caregivers in their immediate environment to be sensitive and to recognise their distress, the urgency of their distress signal, and to take action to decrease it. The parental response not only determines whether pain is identified and whether steps are taken to relieve it, but also shapes the child’s experience and expression of pain [17]. Pain distress is either magnified or moderated by the carers response. A parent experiencing significant anxiety or existential distress associated with an infant’s pain may be less emotionally available to the child during periods of distress [17]. Emotional availability encompasses sensitivity to the child’s cues, and responses that are appropriately non-intrusive, non-hostile and structured to meet their need. Din et al. identified that in a group of infants undergoing vaccination, poor emotional availability is not only associated with increased pain reactivity, but increased pain expression also [18]. Osmun et al. explored this concept further with a similar cohort of infants identifying that over time, caregivers who are consistently emotionally available had infants who learned to better regulate negative emotion around future episodes of pain [19].

Maternal anxiety has independently been shown to reduce sensitivity in interactions with children and their ability to regulate the child’s distress [20]. This sensitivity is vital for assessing the impact of various soothing behaviours and developing a repertoire of individualised soothing techniques for their child.

Positive caregiver behaviours shown to improve distress during pain include vocalisations, proximal soothing such as rocking, stroking, kissing, and sensitivity to the individual preferences of the child as various comforting behaviours by the caregiver are tried [17,20]. Interventions for carers encouraging these positive behaviours should include carer coping strategies such as self-talk and distraction, encouraging positive carer affect, improving carer self-efficacy, and encouraging a sense of control during pain episodes [17,20]. These may improve the emotional availability of the carer to the child with SNI in pain, ameliorating pain related distress for both.

## 3. The Assessment of ‘Total Pain’

Assessing the experience of pain in children with SNI and distinguishing it from other causes of distress is extremely challenging. As a result, pain often goes unrecognised, with only 50% of those with persistent pain receiving analgesia [5,16].

A thorough pain assessment includes a comprehensive history to identify sites of pain, timing, onset, character, associated features, and response to previous pain relief. Table 3 outlines important and often overlooked aspects of the physical examination to identify sources of pain in these children.

It is vital to explore the context, and take into account the psychosocial and spiritual factors discussed above. Impact on sleep and carer response to episodes of pain should be considered. These assessments are best completed using a multi-disciplinary approach, including professionals skilled in psychological and spiritual assessment. In our service, play specialists and hospice family support practitioners, in addition to our chaplaincy service play a vital role in building a picture of the pain experience of our children and families. A period of observation at our local hospice for prolonged assessment and comparison of symptoms outside of the patient’s usual psychosocial context can be extremely helpful. Investigations should be directed by history and examination, co-ordinated by the primary medical team looking after the child. In a palliative population, the appropriateness of invasive investigations should be carefully considered in discussion with the family and all teams involved, with the aim of minimising harm and maximising quality of life for the child and family.

Difficulty encoding expressive behaviour means children with SNI display behaviours that may represent pain or primitive reflexes or abnormal movements. Some display atypical responses to pain, such as sudden stillness (freezing phenomenon), smiling, laughter or self-harming [5]. Parents may misinterpret pain behaviours as part of their usual condition. Despite this, observed pain behaviours are considered a valid approach to assessment of pain in those unable to self-report, and there is now much consensus on pain cues expressed by these patients [2,21]. A myriad of pain tools exist to assess children with SNI. A recent systematic review identified 15 tools of high reliability, validity, comprehensiveness and usability [22]. Of these, three were recommended for children with SNI (Gross Motor Function Classification System Grading IV-V). Two main rating scales were the Paediatric Pain Profile (PPP) and Non-Communicating Children’s Pain Checklist-Revised Version (NCCPC-RV) [23,24]. These both assess a wide range of behaviours, including those pertaining to the psychosocial and spiritual distress associated with the pain experience [22]. A body-map tool is recommended for use alongside these to aid with intensity and location of pain [22]. Tertiary training centres in Paediatric Palliative Care in the UK uniformly use another tool, the Face Legs Activity Cry Consolability (FLACC) tool, revised for children with SNI [25]. Our experience is that using a range of tools enables a more individualised approach to pain assessment. The PPP and NCCPC-RV provide a broader and more information rich assessment of pain during periods of significant instability or diagnostic uncertainty, whereas the FLACC provides a simpler more flexible assessment for contexts and time periods when this is required. A summary of these tools with additional critique can be seen in Table 4.

## 4. An Approach to Managing ‘Total Pain’

Once a thorough assessment of the causes and experience of pain are complete, a holistic, multidisciplinary plan should be put in place to manage the pain, support the family, and monitor and evaluate the impact of interventions.

Management of dystonia and spasticity should be optimised in accordance with national guidance [7,26]. This includes monitoring for hip subluxation and managing orthopaedic complications alongside appropriate specialist teams. For children with undiagnosed pain or abdominal pain, gastro-oesophageal reflux and constipation should be aggressively treated, and the impact of analgesic medications on these symptoms reviewed regularly [5,7]. Bone health and fracture prevention should be considered by maximising mobility, optimising vitamin D and calcium intake, and use of bisphosphonates as required [6]. Medication should be rationalised to limit side effects and interactions which may result in exaggerated symptom experience.

In addition to treatment directed at identified causes of pain, optimal pain management requires a comprehensive approach using opioid, adjuvant and non-pharmacological strategies [27]. Non-pharmacological strategies include swaddling, rocking, repositioning and massage [5,28]. There is limited evidence for the use of music and audiotherapy, acupuncture, aromatherapy, vibratory therapy and weighted blankets in this population [5,28]. Employing the skills of therapists including physiotherapy, occupational therapy and orthotics is vital for children with musculoskeletal pain and disorders of increased tone.

The basis for a logical approach to using opioids in palliative care has been the World Health Organization (WHO) ‘pain ladder’ [21]. In the past, the ladder had three steps: simple analgesia on step 1, ‘weak’ or ‘minor’ opioids on step 2 and ‘strong’ or ‘major’ opioids on step 3. There is, however, no pharmacological basis for making a distinction between ‘weak’ and ‘strong’ opioids. Furthermore, the two most common opioids prescribed on step 2 were codeine and tramadol, both of which are now felt to be potentially hazardous, especially in children [21]. This has led the WHO to describe a two-step analgesia ladder that consists of simple analgesia on the first ‘rung’ and opioids on the second [27]. Hain points out, however, that optimal use of opioids in severe pain is distinctly different from their use in moderate pain. Opioids for severe pain should be given regularly and should be at a higher dose than those for moderate pain. There is therefore still value in separating the use of opioids for moderate pain (Step 2) and that for severe pain (Step 3) [21]. Treatment should be tailored to the individual child, using the least invasive route possible [21]. Additional doses of short acting analgesia should be provided for episodes of predictable (incident) and unpredictable (breakthrough) pain [29].

Central neuropathic causes of pain that play a significant role in children with SNI typically do not respond completely to opioid therapy [5]. Mediations directed at these CNS sources may have a preferential role in children with SNI [5]. Recent guidance by the American Academy of Paediatrics recommends the use of empirical trials of neuropathic agents in children with SNI and persistent pain behaviours. First-line agents include gabapentinoids and tricyclic antidepressants (TCAs), with Clonidine, Methadone, Ketamine and Cannabinoids considered if initial therapy fails [5,30]. Recent Cochrane reviews of antiepileptic and antidepressant medications for non-cancer pain in children found insufficient evidence to formally recommend any of these medications for pain. However, adverse effects with gabapentin, pregabalin and TCAs were uncommon [31]. From adult experimental studies, the number needed to treat to improve pain for TCAs is slightly lower than for gabapentin. However, when quality of life is considered alongside pain severity as an outcome, gabapentin is preferred due to an improved side effect profile [32,33]. From observational paediatric data, gabapentin significantly reduced unexplained irritability related to bowel symptoms in a retrospective observational study of 9 children with SNI [34]. In a further case series, 21 out of 22 children (95%) with SNI demonstrated a significant decrease (greater than 50%) in frequency and severity of pain episodes with gabapentin, with many demonstrating improved feed tolerance [35]. Gabapentin reduces the release of neuroexcitatory neurotransmitters implicated in central neuropathic pain and visceral hyperalgesia. It also inhibits central sensitisation in animals and human studies [32]. Gabapentin is less sedating than TCAs, and may also improve dystonia which so significantly exacerbates pain in many children with SNI [36]. In a retrospective review of 69 children with dystonia, most of whom had SNI, Liow et al. identified significant improvements in sleep, mood, pain, general muscle tone, involuntary muscle contractions and seating tolerance with gabapentin [36]. In light of the above, the authors first choice for an empirical trial would be gabapentin followed by a TCA if treatment failed or was not tolerated. In our experience gabapentin is generally well tolerated, although the sedative effects can be profound in some patients. These patients may benefit from a slower titration to therapeutic doses [5]. While no official dosing guidance exists in this patient group for pain, studies typically started with doses of gabapentin between 2 and 6 mg/kg/day in three divided doses, increasing every few days by 5 mg/kg/day until a response is achieved [5,35]. Therapeutic doses were noted to be between 15–45 mg/kg/day with a maximum suggested dose of 70 mg/kg/day advised by both the American Academy of Pediatrics and National Institute of Health and Care Excellence in the UK [5,37]. Clear goals must be set with families, ensuring a clear plan for dosing, duration of the treatment trial, monitoring, and gradual withdrawal if no improvement is seen [5,30].

## 5. Conclusions

Our understanding of children with SNI, their expression of pain, and the processes underpinning their pain experience have improved dramatically in recent years. The assessment and management of pain in children with SNI, while challenging, is possible with the use of a holistic approach to the physical, psychosocial and spiritual aspects of the pain experience for the child and family. Only by addressing each of these areas in both the assessment and treatment stages, will care teams be able to achieve maximum relief of pain and improvement in quality of life for this patient group.

## Figures and Tables

**Table 1:** Factors contributing to experience of pain in children.

Cognitive Appraisal of Pain [2,3]
Context of disease trajectory [3]
Beliefs about pain [2]
Existential meanings attached to pain [4]
Social abandonment [3]
Anxiety [2]
Depression [2]
Fear of implications of pain on disease [2]
Memories of prior pain [1]
Distress of prior pain [1]
Mental isolation [3]
Boredom [3]
Fatigue [3]
Grieving [3]
Pain tolerance [3]
Coping ability/strategies [3]
Cultural implications of pain and associated functional limitations [2]
Degree of tissue damage
Central excitation and inhibition of afferent signals [1]

**Table 2:** Sources of nociceptive pain in children with severe neurological impairment (SNI).

Common	Less Common
Musculoskeletal (osteopenia, scoliosis, hip subluxation, pathological fractures)	Dental caries
Hypertonia (spasticity, dystonia)	Non-specific back pain
Muscle fatigue and immobility	Renal stones and urinary tract infections (UTI) (topiramate, ketogenic diet)
Constipation	Pancreatitis (valproate and hypothermia)
Gastro-oesophageal reflux disease (GORD)	Cholecystitis (tube feeding)
Gastrointestinal dysmotility (autonomic and post-surgical e.g., fundoplication)	Ventricular shunt blockage, infection
Iatrogenic (investigations, surgery)	Headache [5,7]
Sources common to all children (e.g., Otitis media, dysmenorrhoea, appendicitis)	

**Table 3:** Examination for nociceptive causes of pain in children with SNI.

Eyes—corneal abrasion
Mouth, and throat—dental caries and abscess, gingivitis, tonsillitis
Central lines, implanted devices, shunt catheter sites—malfunction, infection
Gastrostomy tube—gastrostomy tube tension, site infection
Abdomen—constipation, distention
Skin—hair tourniquet or pressure ulcer
Extremities and joints—occult fracture, subluxation [5]

**Table 4:** Pain assessment tools in children with severe neurological impairment (SNI).

Tool Name	Description	Process of Validation	Key Interpretation	Positive Features	Negative Features
Paediatric Pain Profile (PPP) [23]	20 Item behaviour scale.	Interviews 21 children, 26 professionals + Questionnaire 121 parents to develop scale. Children with severe cognitive impairment.	Robustly developed and validated in real world setting.	Designed specifically for non-verbal children with SNI.	Lengthy compared to FLACC scale.
	Four-point scale for each item.	Interrater reliability acceptable for combined item score.	Clear difference in pain scores between when in pain and no pain with narrow confidence interval.	Can compare scores with a baseline score. Completed in 2–3 min.	
	Assess pain at baseline, then repeatedly for regular monitoring or to monitor intervention.	Correlation between raters of 0.75.	Reliable between raters.	Descriptors of more than one pain type.	Many behaviours open to significant interpretation.
	Designed to be parent held.	Sensitivity of 1.0 and Specificity of 0.91 for moderate/severe pain at PPP score of 14.	Very sensitive and specific for detecting pain.	Takes into account psychosocial aspects.	
Face Legs Activity Cry Consolability (FLACC) [25]	Five-items behaviour scale.	Validated on children with varying degrees neurological impairment.	Small sample size of 50 patients in validation study.	Can add individual behaviours to the pain scale for each child.	Validated in a post-surgical population only.
	Three-point scale per item (0–2).				
	Option for individualised items to be included.	Validated in 52 children with 80 observations per-operatively including video assessment by experts.		Quick, un-ambiguous tool to use.	
	Mild pain = 0–3.		Correlations of scores to parental perceived pain good, but cut-offs defined for mild/moderate/severe are not validated.		Fewer behaviours assessed so data less rich to inform assessment.
	Moderate pain = 4–6.	Good inter-rater correlations of total score (0.90 (0.87–0.92)) between nurse observations.		Very high interrater correlations of total score.	
	Severe pain = 7–10.				
Non-Communicating Children’s Pain Checklist—Revised [24]	30 items behaviour scale.	71 children assessed. Daily 2 h observation for 1 week. Repeated 3 monthly.	Thorough validation of pain tool.	Many behaviours assessed over long assessment period so rich data for pain assessment.	2 h observation period may be impractical for many carers.
	Four-point scale per item (0–4).	Inter-rater correlation for total score of 0.46, statistically significant but not strong correlation. Correlation between numerical pain score of parent and pain scale was 0.64 and for researcher and parent pain score 0.72.	High specificity and sensitivity at score cut-off.		
	2 h observation period required per scoring.	Score of 7 or greater had Sensitivity 84% and Specificity 68% for pain.	Weaker correlations between raters and parent rating than other scales.	Behaviours clearly described and unambiguous.	
